# A study of the opinions of Swedish healthcare personnel regarding acceptable outcome following decompressive hemicraniectomy for ischaemic stroke

**DOI:** 10.1007/s00701-017-3358-y

**Published:** 2017-11-04

**Authors:** Magnus Olivecrona, Stephen Honeybul

**Affiliations:** 1 Department of Anaesthesia and Intensive Care, Section for Neurosurgery, Faculty of Health and Medicine, Department for Medical Sciences, Örebro University, Örebro, Sweden; 2 Departement of Neurosurgery, Sir Charles Gairdner Hospital, Perth, WA Australia

**Keywords:** Malignant middle cerebral artery infarction, Hemicraniectomy, Outcome, Ethics

## Abstract

**Background:**

Decompressive hemicraniectomy (DC) is an established lifesaving treatment for malignant infarction of the middle cerebral artery (mMCAI). However, surgical decompression will not reverse the effects of the stroke and many survivors will be left severely disabled. The objective of this study was to assess what neurological outcome would be considered acceptable in these circumstances amongst Swedish healthcare workers.

**Method:**

Healthcare workers were invited to participate in a presentation that outlined the pathophysiology of mMCAI, the rationale behind DC and outcome data from randomised controlled trials that have investigated efficacy of the procedure. They were then asked which neurological outcome would they feel to be acceptable based on the modified Rankin Score (mRS) and the Aphasia Handicap Scale (AHS). Information regarding sex, age, marital status, relatives, religion, earlier experience of stroke and occupation was also collected.

**Results:**

Six hundred and nine persons participated. The median accepted mRS was 3. An mRS of 4 or 5 was perceived to be acceptable by only 30.5% of participants. Therefore the most likely outcome, based on the results of the randomised controlled trials, would be unacceptable to most of the participants [OR 0.39 (CI, 0.22–0.69)]. The median accepted AHS was 3. A worst language outcome of restricted autonomy of verbal communication (AHS 3) or better would be accepted by 44.6%.

**Conclusions:**

This study has highlighted the ethical problems when obtaining consent for DC following mMCAI, because for many of the participants the most likely neurological outcome would be deemed unacceptable. These issues need to be considered prior to surgical intervention and the time may have come for a broader societal discussion regarding the value of a procedure that converts death into survival with severe disability given the attendant financial and healthcare resource implications.

## Introduction

The past decade has seen considerable advances in the management of ischaemic stroke and a number of clinical trials have demonstrated the significant reduction in mortality and improvement in outcome that can be achieved by endovascular techniques using either intra-arterial therapy or mechanical thrombectomy [1, 5, 9]. However, the time-dependent nature of these interventions means that there will always be patients who either present outside the therapeutic window or for whom endovascular therapy fails.

Approximately 1–10% of these patients will go on to develop life-threatening cerebral oedema, so called “malignant” middle cerebral artery infarction (mMCAI) and the prognosis for these patients is poor with a mortality rate in the region of 80%. In these circumstances, consideration may be given to decompressive craniectomy as a lifesaving intervention. One of the first descriptions of the technique was from Professor Koch in 1905 and he asserted that “if there is no CSF pressure, but brain pressure exists, then pressure relief must be achieved by opening the skull” [19]. The rationale for surgical decompression is that death due to tonsillar herniation is prevented and the reduction in mortality has been clearly demonstrated by recent randomised controlled trials [10, 17, 18, 25, 26]. However, unlike the endovascular techniques, surgical decompression will not reverse the effects of what is by definition a very extensive infarct and many patients will be left with significant neurological deficits [15, 24]. Indeed, in the pooled analysis of the three European stroke trials, it was only possible to conclude that surgery improved clinical outcome by redefining the favourable category such that it included patients with a modified Rankin Scale (mRS) of 4 [25]. Favourable outcome would, therefore, include patients who could not walk unaided, could not look after their bodily needs and were, therefore, dependent. To justify this recategorisation, the authors have stated that “On the basis of increasing experience of long-term outcome in patients with a space-occupying infarction, most investigators feel obliged to define a score of 4 on the modified Rankin scale (mRS) as favourable”. However, closer examination of the literature shows little evidence to support this statement [13].

The aim of this study was to canvass opinion amongst healthcare workers in Sweden regarding the acceptable outcome in terms of mRS and assess opinion regarding the importance of language preservation.

## Methods

Staff in the Departments of Neurology, Anaesthesiology and Intensive Care (ANICU) and Cardiology at the University Hospital in Örebro, Sweden were invited to participate in the study during one of their annual education days. Participants were presented with information regarding the pathophysiology of mMCAI and the expected mortality if untreated. This included an introduction to basic concepts such as the Monroe Kellie doctrine and the role of DC as a lifesaving intervention. It was emphasised that surgery would not reverse the effects of the stroke but would certainly reduce mortality. The results of recent randomised controlled trials were presented in order to inform participants of the most likely outcome following surgical intervention when compared to standard medical therapy. The need for informed consent and the regulatory requirements in Sweden was also was presented. The aim of the presentation was to provide unbiased evidence regarding outcome in a similar manner to which this type of information is conveyed to patients and their relatives. After the presentation, participants were given the opportunity to ask questions. They were then asked which functional level in terms of mRS they would accept if they were to survive following DC for mMCAI (Table 1) [8].

**Table 1:** Modified Rankin Scale [9]

Score	Description
0	*No symptoms at all*
1	*No significant disability despite symptoms*; able to carry out all usual duties and activities
2	*Slight disability*; unable to carry out all previous activities, but able to look after own affairs without assistance
3	*Moderate disability*; requiring some help but able to walk without assistance
4	*Moderately severe disability*; unable to walk without assistance and unable to attend to own bodily needs without assistance.
5	*Severe disability*; bedridden, incontinent and requiring constant nursing care and attention
6	*Death*

The questionnaire was similar to that used by Honeybul et al. [14] in a previous study. (Questionnaire available as supplemental data.) There were modifications in that there were additional questions concerning the acceptability of language deficits according to the Aphasia Handicap Scale (AHS), ranging from 0 to 5 (Table 2) [2]. Additional information regarding sex, age, marital status, relatives, religion, earlier experience of stroke and occupation was also collected.

**Table 2:** Aphasia Handicap Scale [2]

Score	Description
0	*Normal language*
1	*Minor difficulties* of language without disability (no impact on normal life)
2	*Mild language-related disability* (without restrictions in the autonomy of verbal communication in daily life)
3	*Moderate language-related disability* (restricted autonomy of verbal communication)
4	*Severe language-related disability* (lack of effective verbal communication)
5	*Mutism or total loss* of verbal expression and comprehension

### Statistics

Continuous and categorical data are presented as numbers and percentages and interquartile range. Differences between groups were analysed by χ^2^ tests, as indicated. The statistical analysis was performed with JMP ver. 12 (SAS Institute, Cary, NC, USA). A *p* value of <0.05 was considered as statistically significant.

### Ethics

The Regional Ethical Review Board in Uppsala has decided that the study does not need ethical review (Dnr 2014/451).

## Results

Six hundred and nine persons answered the questionnaire. The characteristics of the persons are presented in Table 3.

**Table 3:** Characteristics of the 609 participants

Variable (*n* = number of answers)		*n* (%)
Sex (*n* = 603)	Female	505 (83.2)
	Male	98 (16.2)
Age groups, years (*n* = 601)	18–25	24 (4)
	25–35	107 (17.8)
	35–45	139 (23.1)
	45–55	170 (28.3)
	55–65	154 (25.6)
	65-	7 (1.2)
Marital status (*n* = 606)	Married / common-law partner	462 (76.2)
	Single	108 (17.8)
	Divorced	36 (5.9)
Relatives (*n* = 597)	None	14 (2.3)
	Only children	71 (11.9)
	Only parents	16 (2.7)
	Only Siblings	9 (1.5)
	More than one category	487 (81.6)
Religion (*n* = 589)	Christianity	312 (53.0)
	None	238 (40.4)
	Will not state	18 (3.1)
	Islam	9 (1.5)
Experience of stroke (*n* = 601)	None	42 (7.0)
	As a healthcare worker	433 (72.0)
	Relative	253 (42.1)
	Acquaintance	154 (25.6)
Profession (*n* = 571)	Floor nurse	112 (19.6)
	Registered nurse	338 (59.2)
	Physician	74 (13.0)
	Physiotherapist	18 (3.2)
	Other	29 (5.1)

**Fig. 1:**
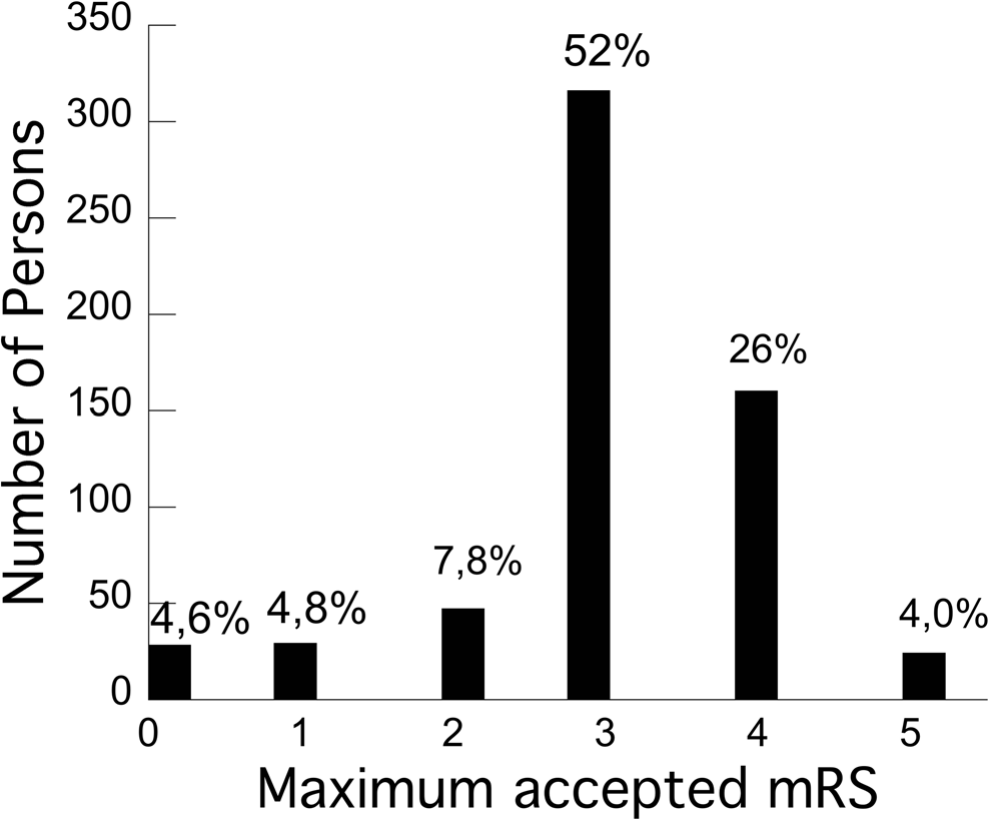
Distribution of worst accepted functional outcome after decompressive craniectomy for malignant media infarction, measured as mRS

**Fig. 2:**
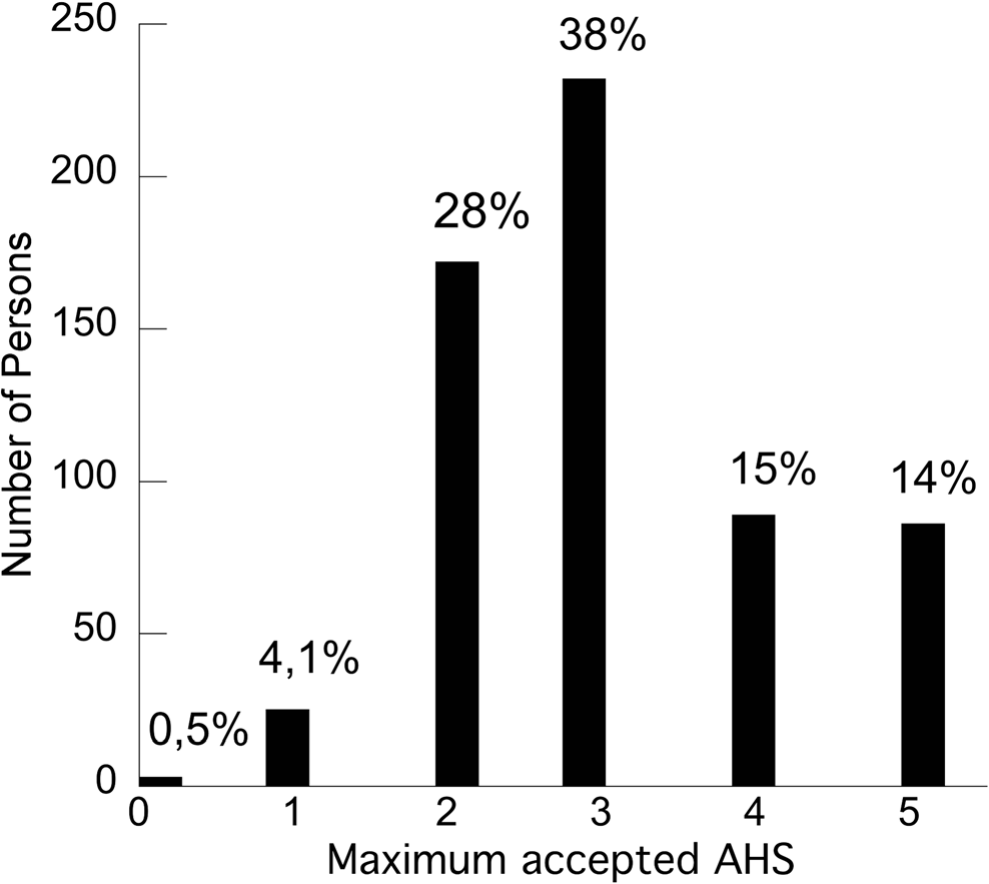
Distribution of worst accepted outcome in regard to language disturbance after decompressive craniectomy for malignant media infarction, measured as AHS

The distribution of the highest accepted mRS (worst function) is illustrated in Fig. 1. The median acceptable mRS was 3 (IQR 1). The distribution of the highest accepted (worst function) AHS is illustrated in Fig. 2. The median acceptable AHS was 3 (IQR 2).

There was significant difference between the sexes in regard to the worst accepted functional outcome (mRS) (Pearson’s χ^2^, *p* < 0.05). Males seemed to accept a worse functional outcome compared to the females. There was no significant difference between the sexes with regard to accepted language disturbance (Pearson’s χ^2^, *p* = 0.07). No significant difference was found in analysing accepted worse outcome of mRS or AHS in relation to age, marital status, relatives, religion, earlier experience of stroke, profession or department.

Analysing the dichotomisation of mRS in 0–3 [*n* = 420 (69.5%)] and 4–5 (*n* = 184) significantly more participants would accept a mRS of 3 compared with those who would accept an outcome of mRS 4–5 (χ^2^, *p* < 0.001). There were no significant differences with regard to sex, age, marital status, relatives, religion, earlier experience of stroke, profession, or department.

The odds ratio (OR) for the investigated population to reach the most likely outcome based on the results of the three randomised studies (excluding mortality as the question asked in the study specified survival) (*see* Table 4). The OR was 0.39 (95% CI, 0.22–0.69) and the number needed to harm 3.1.

**Table 4:** Results as mRS in the three randomised trials

	mRS 2	mRS 3	mRS 4	mRS 5	mRS 6	Total
Jüttler et al. [19]	4	4	5	1	3	17
Vahedie et al. [28]	3	7	5	0	5	20
Hofmeijer et al. [11]	1	7	11	6	7	32
Total	8	18	21	7	15	69
mRS ≤ 3	26					
mRS ≤ 4	47					

If the dichotomisation was made between mRS 0–4 (*n* = 580) and mRS 5 (*n* = 24), there is was significant difference amongst the sexes. Males accepted an mRS of 5 more often than females (Fisher’s exact test *p* < 0.05). There was no significant difference in terms of age, marital status, relatives, religion, earlier experience of stroke or profession.

Doing the dichotomisation of AHS in 0–3 (*n* = 432) and 4–5 (*n* = 175), a significant difference is found between the sexes. Males would accept a worse AHS score than females (Fisher’s exact test, 0.005). There was no significant difference found between the two groups with regards; age, marital status, relatives, religion, earlier experience of stroke, profession or department. When dichotomising between AHS of 0–4 (*n* = 521) and 5 (*n* = 86), the same significant difference was found between the sexes (Fisher’s exact test, 0.27); however, no other significant differences were found.

## Discussion

The results of the recently published randomised controlled trials clearly demonstrate that surgical decompression significantly reduces mortality; however, this results in an almost direct translation into the number of survivors with a mRS of 4 [10, 17, 25, 26]. Whilst the investigators in these studies changed the outcome dichotomy such that this outcome was deemed to be favourable, the results of this study would call this recatagorisation into question.

Indeed, based on the results of the trials and the responses of participants in this study, approximately every third person who survives following a DC for mMCAI (risk to harm 3.1) would do so with a level of disability that beforehand they would have felt to be unacceptable.

These findings are in concordance with a similar investigation performed amongst Australian healthcare workers in which only 8.7% of participants regarded an mRS of 4–5 as acceptable [14]. There were similar finding in a survey that assessed opinion in an online survey amongst North American and Asian neurologists, neurosurgeons and neurointensivists [22], and in a study that investigated opinion in patients who had previously suffered a stroke (and presumably thereby has first-hand experience of the consequences of stroke). Most respondents felt that survival with dependency (mRS of 4–5) would be unacceptable [23].

Overall, the results of these studies, in conjunction with the current study, would appear to provide compelling evidence that considering an mRS of ≥4 as acceptable must at the very least be called into question or perhaps recategorised back to unacceptable. However, even if this were to occur, there remain questions regarding the ongoing use of DC given the clear reduction in mortality and there are a number of issues that require consideration.

### Consent

Informed consent forms one of the fundamental tenets of modern medicine. This requires that a competent person, given a clear understanding of the facts, implications and future consequences of an action, makes a decision prior to any intervention based on what they perceive to be an acceptable outcome, given their personal values [3, 4, 6]. In the context of a clinical deterioration following an acute stroke these requirements are challenged because the patient will be in no position to make competent judgements regarding their healthcare preferences and in these circumstances family members are often called upon to provide support for the decision to surgically intervene. This can place enormous psychological burden on relatives who are already distressed due to the impact of the initial illness and it is in this regard that the results of this type of study may be useful, not least because it may promote discussion in the wider community regarding these issues.

Although it could of course be argued that the circumstances in which participants were placed represented a relatively hypothetical situation that is not truly representative of the real live ethical tension that occurs in the context of an acute hemispheric stroke. It could equally be argued that in the real life clinical setting decisions have to be made under pressure of limited time and perhaps limited competency (due to emotional distress) to fully reflect on the long-term implications.

There is, however, no doubt that the circumstances in which participants were placed fulfils the criteria for truly informed consent, in that the implications of the intervention and likely outcome were fully explained and were competent to make an assessment regarding what they would feel to be an acceptable outcome [6, 7]. Notwithstanding some limitations when making somewhat abstract observations such as “I would never want to live with severe disability”, this type of assessment forms the basis of documentation such as living wills and advance directives. If a person has previously made their wishes known, either voiced or documented, regarding the acceptability or otherwise of survival with dependency these wishes should be acknowledged and where possible acted upon.

Based on this study, it would appear problematic to include an mRS of 4 within the outcome category of favourable and considerable caution must be exercised when this is going to be the most likely outcome (such as in patients over 60 years of age).

### Retrospective consent

It must be acknowledged that humans are highly adaptable and can learn to live with a level of disability that they might previously thought to be unacceptable. Studies that have investigated the issue of retrospective consent provide support for this position. Indeed, the authors of the pooled analysis of the three European trials, justified the inclusion of an mRS of 4 within the favourable outcome category, because they obtained positive responses when they asked survivors whether they regretted having had the surgery, given they had survived, but remained disabled [25].

Furthermore, it would be difficult to state that it has not been in a person’s best interests if they survive a neurological catastrophe but are able to state that they do not regret the intervention given their eventual quality of life. However, accepting this as a variation of the consenting process and, therefore, justifying the surgical procedure is not only ethically problematic but it also challenges the aforementioned necessity of informed consent prior to medical intervention. A more realistic interpretation of these responses is that these patients may have adapted to a level of disability that they might previously have deemed to be unacceptable and “recalibrated” their lifestyle expectations [16, 20].

A further consideration is that not all studies have obtained such high levels of retrospective consent, especially amongst the elderly. A number of studies have reported high levels of anxiety and depression, especially amongst survivors who have been left dependent [11, 21]. The recent DESTINY II trial that investigated efficacy of DC in patients over 60 years of age reinforced this observation [18]. Notwithstanding the investigators conclusion that “hemicraniectomy increased survival without severe disability”, most survivors were dependent and unable to communicate. Amongst those patients randomised to the surgical arm of the trial, there were 27 survivors. Two patients of those achieved an mRS score of 3. Of the remaining 25 patients, there was an equal distribution of patients between an mRS of 4 and 5, and 16 of these patients had such severe aphasia or neuropsychological deficits that they were unable to provide a response regarding retrospective consent [17, 20].

These findings have in many ways undermined the legitimacy of retrospective consent and served to emphasise the need for significant discussion to occur prior to surgical intervention especially for persons in this age group [17].

### Language preservation

The issue of language preservation and surgical decompression in the context of dominant hemispheric stroke has been a source of debate for many years. A surprising finding in this study is that 67% of participants would accept restricted verbal communication (i.e. AHS 3 or worse).

This is a similar finding to that of a previous study that has investigated opinion amongst stroke victims and their families, where it was found that amongst patients who had previously had a stroke, 52% would agree to a DC if the mMCAI was in the dominant hemisphere [23]. However, the interesting and perhaps more surprising finding is that if the mMCAI was in the non-dominant hemisphere only 42% would agree to have a DC. It is difficult to know how these findings should be interpreted, and it may be that language function and communication are not felt to as important as has previously been thought. The problem will always be how to determine the acceptability or otherwise of survival with severe speech difficulties as patients are often unable or have difficulty communicating their preferences. This was clearly demonstrated in the DESTINY II trial, in which most survivors could not answer a simple question regarding retrospective consent and would not therefore be in position to discuss more complex issues regarding quality of life.

### Cultural issues and possible bias

Finally, this study has demonstrated the wide range of responses that individuals provide and highlighted possible cultural differences that require consideration. In the current study, approximately 30% of participants within the Swedish healthcare system felt that survival with an mRS of 4 to be acceptable. Amongst Australian healthcare workers, only 10% would accept this outcome [14]. These differences may be based on current clinical practice within the particular healthcare environment in which the participants were involved or on socioeconomic factors. Both the current study and the Australian study failed to demonstrate any difference in responses based on religious orientation; however, there may be more subtle cultural influences and this would be an interesting aspect to explore further.

It must of course be acknowledged that there is potential for personal bias to influence the manner in which the data are presented and therefore interpreted by the participants. The senior author of the Australian study (S.H.) has frequently expressed a view that survival with an mRS of 4 should be categorised as unfavourable and this may (even unintentionally) have influenced the participants to give a negative response regarding this outcome [12, 15]. Alternatively, the higher response in the current study may reflect cultural values intrinsic to the Swedish society where survival at any cost may be felt to be important.

## Conclusions

Overall there will never be a one-size-fits-all approach to the difficult ethical issues that require consideration when considering an intervention that potentially converts death into survival with dependency. The decision to surgically intervene requires consideration not only of personal preferences but also regarding the significant health resource implications that will arise whether it be in the context of ischemic stroke or for other neurological catastrophes. The time may have come for a broader discussion regarding the value placed on survival for any one individual at any cost, and the considerations here are not only financial but also ethical and moral regarding sustainable and equitable allocation of scarce healthcare resources.
